# Considerations for Applying Metabolomics to the Analysis of Extracellular Vesicles

**DOI:** 10.3389/fimmu.2014.00651

**Published:** 2014-12-23

**Authors:** Laura Palomo, Enriqueta Casal, Felix Royo, Diana Cabrera, Sebastiaan van-Liempd, Juan M. Falcon-Perez

**Affiliations:** ^1^Metabolomics Unit and Platform, Center for Cooperative Research in Biosciences (CIC bioGUNE), Derio, Spain; ^2^Centro de Investigacion Biomedica en Red de Enfermedades Hepaticas y Digestivas (CIBERehd), Madrid, Spain; ^3^Ikerbasque, Basque Foundation for Science, Bilbao, Spain

**Keywords:** extracellular vesicles, exosomes, microvesicles, metabolomics, metabonomics, protocol standardization

Cell-derived extracellular vesicles (EVs) are involved in the development of different pathologies including inflammatory diseases and cancer (1) and have demonstrated a promising potential for human diagnostic (2) and therapeutic applications (3). Constitutive secretion of these vesicles has been described for platelets, reticulocytes, dendritic cells, lymphocytes, mast cells, intestinal epithelial cells, adipocytes, and hepatocytes among others (4). In addition, EVs have been isolated from many body fluids including bile (5), blood (6), and urine (7, 8), which indicates that they can be systemically disseminated, transferring their content/signals to cells physically separated from the secreting cell. Depending mainly on the vesicle’s origin and the way of vesicle-discharge from the cells, at least two types of EVs have been described: the endosome-derived vesicles named “exosomes” and the plasma membrane shedding vesicles referred to as microparticles. Microparticles are a heterogeneous population of vesicles with a size up 1000 nm, this group consists of vesicles that are formed directly from plasma membrane by so-called reverse budding through membrane protrusion and fission (9). Exosomes, on the other hand, are a more homogeneous vesicle population with a size of 30–150 nm and an endocytic origin. They are formed by inward budding of the membrane of an endocytic organelle named multivesicular body and released to the extracellular space by fusion of this organelle with the plasma membrane (9).

The content of EVs and their biological function depend on the cell-type origin. In addition to lipids (10, 11) and proteins (10, 11), EVs give also refuge to mRNA, small RNAs including miRNA (12–17), mtDNA (18), and even genomic DNA (19). It is important to highlight that this material could be incorporated during the budding process of EVs in which a portion of cytosol is also engulfed into the vesicles (20) by controlled mechanisms (21, 22). In the process of their formation, cytosolic small molecules (metabolites) such as sugars, amino acids, nucleotides, different enzymatic cofactors, or redox regulatory molecules among others are also included into the vesicles. However, data regarding metabolites associated with EVs are still very limited and therefore extensive work in this is necessary in this area. This research effort is not only needed to map the metabolome of EVs from different origins but also to elucidate whether there are specific mechanisms at play for loading predestined metabolites into specific vesicles.

Extracellular vesicles are widely studied in order to better understand their physiological and pathological role as well as to identify potential non-invasive biomarkers (23). Recent research indicates that EVs have an important effect on the development and progression of diseases such as cancer (24) or immunological (25) diseases. Some of the mechanisms of action responsible for these effects are starting to be unraveled. Remarkably, some publications have demonstrated the involvement of EVs in the metabolic regulation of the extracellular space. In this context, EVs derived from cancer cells are able to modify and educate the microenvironment to facilitate tumor growth and the establishment of metastasis (26, 27). The importance of EVs in keeping the normal homeostasis in the neuronal environment is demonstrated by the implication of EVs in the development of Alzheimer and prion-related diseases (28). Clayton and collaborators have showed that EVs modify the extracellular adenosine levels, which has important implications for the activation of the immune system (29). Taking into account the fact that EVs from different cellular origins and with different activities can co-exist in a determined environment and condition, it is clear that these recent studies only reveal the “tip of the Iceberg.” It is therefore important to elucidate the function of EVs in other cellular systems to understand the complex EV network, which will influence the final outcome of a determined stimulus or biological process. In the case of hepatocyte-derived EVs, a comprehensive proteomic analysis revealed the presence of proteins involved in metabolizing lipoproteins, endogenous compounds, and xenobiotics, which suggests a role of exosomes in the metabolism of these molecules (30). Our proteomics analysis identified more than 100 different proteins with the potential to modify the extracellular space. These include glycolytic enzymes, fatty acid modifying-enzymes, carboxylesterases, cytochrome P450 monooxygenases (CYPs), and uridine dinucleotide phosphate glucuronosyl transferases (UGTs), among others. The fact that hepatocytes are able to secrete EVs that contain a high number of enzymes to extracellular compartments could also suggest that hepatocyte-derived EVs may play a role in the homeostasis of biofluids including blood and bile (31, 32).

To elucidate both the metabolome of EVs and the contribution of these vesicles to hepatic and extra-hepatic metabolism, comprehensive technological platforms are needed to integrate the final outcome of the various activities of these vesicles. In this context, the last-up – omic technology, referred as metabonomics or metabolomics, has opened new opportunities to provide a global view of mechanisms and pathways involved in normal physiological processes as well as in the development of diseases. Metabolomics comprises the qualitative and quantitative measurement of the metabolic response to physiological or pathological stimuli. It involves the extraction and measurement of hundreds to thousands of small molecules (<2000 Da) from cells, tissues, or biofluids to generate metabolic profiles (33). Comparisons of such profiles from different genotypes are being used to identify specific metabolic changes leading to the understanding of physiology, toxicology, and disease progression. The recent developments in spectroscopic and separation methods allow for quick and simultaneous measurements of all classes of metabolites in biological samples (33, 34). Advanced bioinformatics and biostatistics can then be used for data mining and modeling. Metabolomic profiling can be performed using a number of analytical techniques including high-field nuclear magnetic resonance (NMR), gas chromatography/mass spectrometry (GC/MS), and liquid chromatography/MS (LC/MS). Despite its excellent inter-lab reproducibility, GCMS requires that the majority of metabolites analyzed need derivatization to provide volatility and thermal stability prior to analysis (35). While NMR demonstrates its advantages in highly selective, quantitative and non-destructive analysis, the sensitivity and therefore the amount of detected metabolites is low compared to MS. LC/MS, while notoriously irreproducible, possesses much higher sensitivity toward most metabolic classes than NMR. Moreover, by using tandem MS in combination with high-resolution spectra, isotope distributions, and ion mobility, identification of unknown metabolites is facilitated. However, due to the before-mentioned irreproducibility chemical standards are normally necessary for absolute metabolite identification. The development of ultra-performance liquid chromatography (UPLC) has made it possible to achieve higher resolutions, higher sensitivities, and rapid separations as compared to those achieved using conventional LC (36).

Moreover, the combination of metabolomics and specialized software devoted to visualize cellular pathways offer a tool to integrate and identify the main mechanisms that trigger a specific biological process. Application of metabolomics is growing rapidly in an increasing range of fields such as biomarker discovery, clinical studies, diagnostics, plants, nutrition, and toxicology (37, 38). Furthermore, metabolic biomarkers are expected to be less species dependent than gene or protein markers, facilitating the direct comparison of animal models with human studies (39).

Form a practical point of view, it is important to highlight that for the characterization of the metabolome of EVs additional controls are required that are currently not included in the proteomics and transcriptomics analysis of these vesicles. This is exemplified by the UPLC-MS/MS analysis of exosomes-depleted media that has been incubated in the absence of cells during 72-h at 37°C, and subjected to the conventional ultracentrifugation method currently used to isolate EVs (40). In these control samples, substantial number of metabolites were detected using conventional solvents to extract metabolites (Figure 1A: ClCH_3_:methanol, Figure 1B: methanol). These metabolites are derived from the tissue culture medium and need to be taking into account and removed from the metabolites detected in the medium that has been conditioned by the cells in order to identify the metabolome truly associated with the EVs of interest. In the case of proteomics and transcriptomics analyses, the background introduced by the tissue culture media can be overcome given that currently tissue culture media used serum from bovine origin as major contributor to the background. Given the species-specific features of proteins and nucleic acids from the media, the contaminating material can be discriminated from the EVs secreted by the cells of interest. An exemption to this latter has to be made if a cell line of bovine origin is used for the production of EVs; in that case a cell free medium incubated under the same conditions is also recommended for proteomics and transcriptomics analyses.

**Figure 1:**
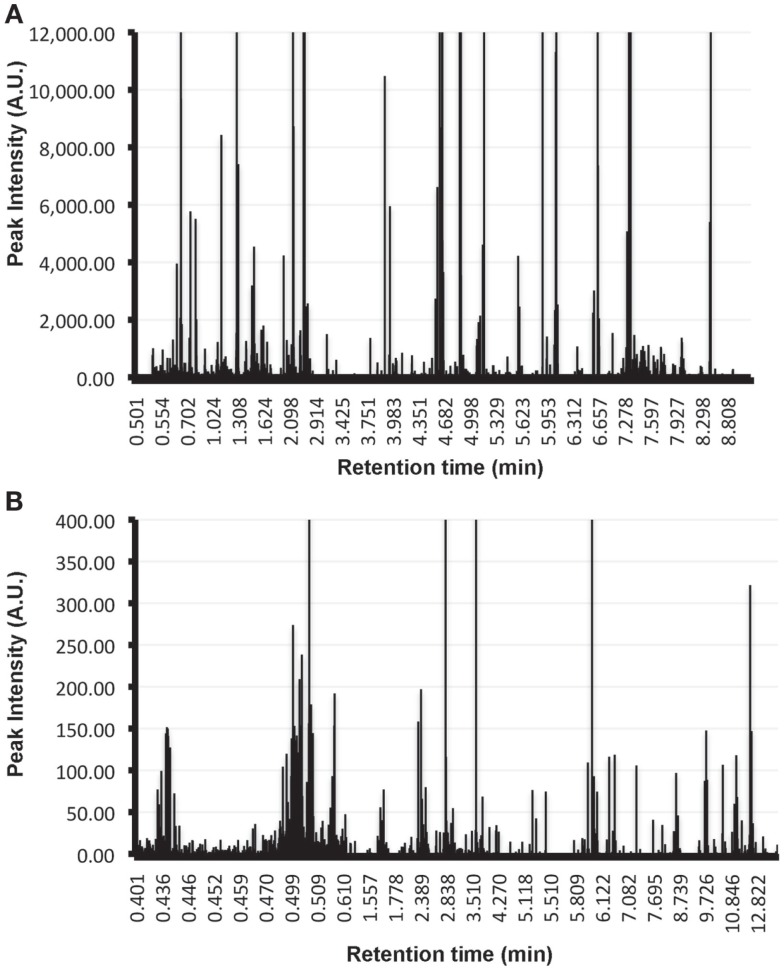
**Metabolic background introduced by tissue culture media in EVs analysis**. Metabolites were extracted using Cl_3_Cl:methanol **(A)** or methanol alone **(B)** from the pellet obtained by applying the conventional ultracentrifugation-based exosomal purification procedure to 300 ml of exosomes-depleted complete media incubated at 37°C during 72 h in absence of cells. Extracted metabolites were analyzed by UPLC-MS/MS using a C18 chromatographic column. Retention time of the metabolites along with their peak intensities are indicated in the plots. Note the elevated number of metabolites of different chemical nature that are isolated using the exosomal purification procedure and that need to be removed in order to determine the metabolome that is truly associated to EVs.

## Conflict of Interest Statement

The authors declare that the research was conducted in the absence of any commercial or financial relationships that could be construed as a potential conflict of interest.
